# An Enzymatic Platform for the Synthesis of Isoprenoid Precursors

**DOI:** 10.1371/journal.pone.0105594

**Published:** 2014-08-25

**Authors:** Sofia B. Rodriguez, Thomas S. Leyh

**Affiliations:** The Department of Microbiology & Immunology, The Albert Einstein College of Medicine, Bronx, New York, United States of America; Virginia Tech, United States of America

## Abstract

The isoprenoid family of compounds is estimated to contain ∼65,000 unique structures including medicines, fragrances, and biofuels. Due to their structural complexity, many isoprenoids can only be obtained by extraction from natural sources, an inherently risky and costly process. Consequently, the biotechnology industry is attempting to genetically engineer microorganisms that can produce isoprenoid-based drugs and fuels on a commercial scale. Isoprenoid backbones are constructed from two, five-carbon building blocks, isopentenyl 5-pyrophosphate and dimethylallyl 5-pyrophosphate, which are end-products of either the mevalonate or non-mevalonate pathways. By linking the HMG-CoA reductase pathway (which produces mevalonate) to the mevalonate pathway, these building block can be synthesized enzymatically from acetate, ATP, NAD(P)H and CoA. Here, the enzymes in these pathways are used to produce pathway intermediates and end-products in single-pot reactions and in remarkably high yield, ∼85%. A strategy for the regio-specific incorporation of isotopes into isoprenoid backbones is developed and used to synthesize a series of isotopomers of diphosphomevalonate, the immediate end-product of the mevalonate pathway. The enzymatic system is shown to be robust and capable of producing quantities of product in aqueous solutions that meet or exceed the highest levels achieved using genetically engineered organisms in high-density fermentation.

## Introduction

Improved access to large numbers of pure proteins, and a rapidly increasing repertoire of well characterized enzymes, isoenzymes and mutants have substantially increased the potential to utilize *in situ* metabolic pathways, or concatenated enzymatic reactions, in the synthesis of complex natural and synthetic products. Enzymes have been honed over evolutionary time to accomplish specific catalytic tasks [1], [2]. Many are extremely efficient, regio-selective catalysts, while others exhibit broad substrate specificities that can provide flexibility in synthetic schemes. Indeed, significant efforts are underway to develop enzymes whose catalytic properties have been altered to achieve specific synthetic goals [3]–[5]. Enzymatic synthesis has been used to produce numerous valuable compounds [6]–[16] and often provides significant enhancements in yield, purity, production time and cost when compared to traditional chemical synthetic methods [17], [18]. Considerable effort is being expended to develop cell-free enzymatic systems for the production of biofuels, including dihydrogen [19] and butanol [20], biomass conversion to starch [21], and high-energy-density biobatteries [22]. While enzymatic synthesis will never replace traditional synthesis, it provides a valuable adjunct to traditional approaches particularly when the objective is to build complex natural products.

The medicinal values of isoprenoids have been documented as early as 168 BC [23], [24]. Today, we are only beginning to understand the social and commercial potential of this enormous, diverse family of natural compounds, which is estimated to contain approximately 65,000 unique structures [25]. Biotechnology companies are attempting to synthesize isopreonoid-based medicines, cosmetics [26], flavors [27], fragrances [28] and biofuels [29]–[31] by genetically engineering plants and bacteria to produce desired isoprenoids in commercial quantities [29], [32]–[34]. Recent efforts along these lines include attempts to genetically engineer organisms to produce artemesinin (an antimalarial) at costs that will significantly expand third-world access to this drug [35],[36], and to produce isoprenoid-based fuels [30], [31].

**Figure 1:**
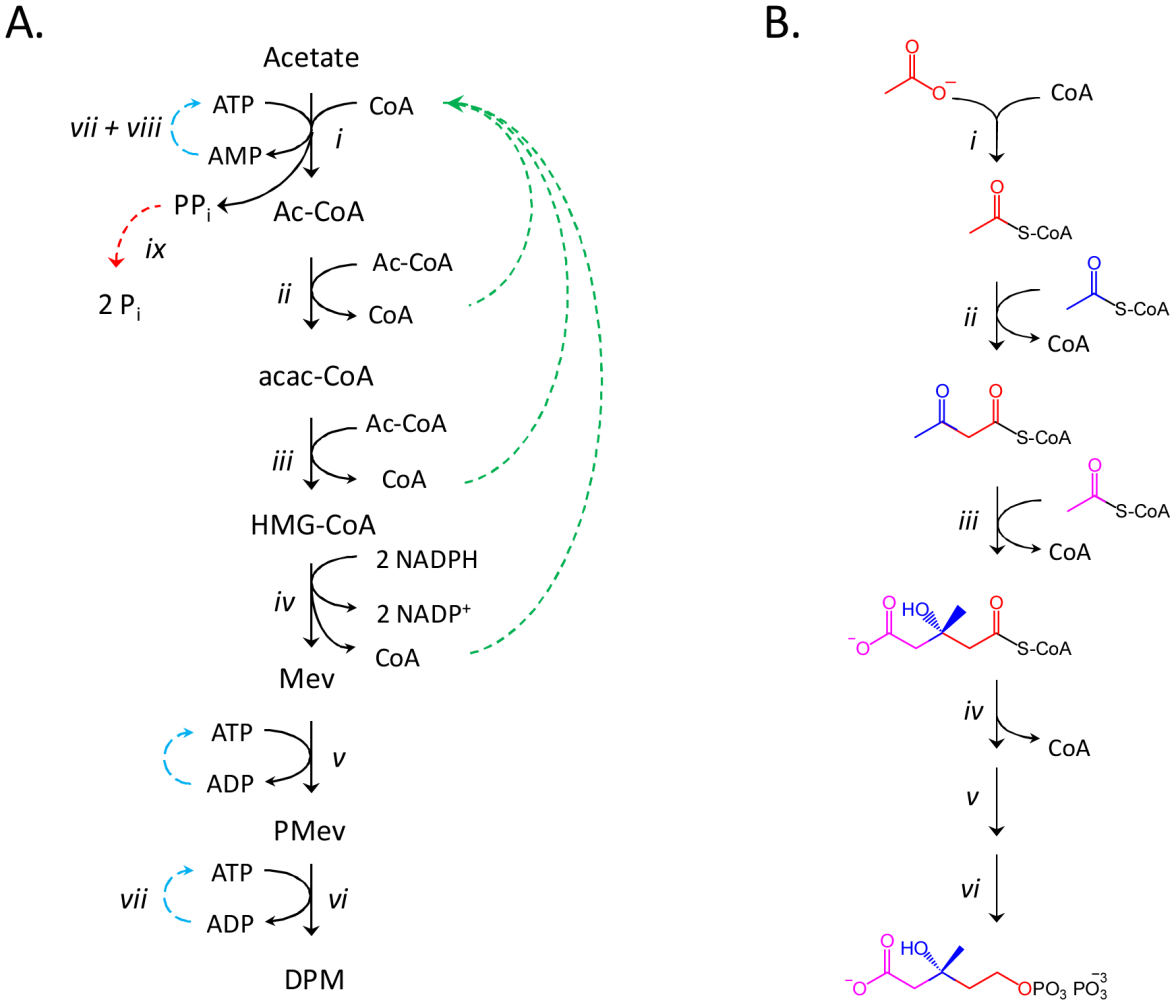
Schematics for the in-situ enzymatic synthesis of DPM and its isotopomers. **Panel A.** The enzymatic synthesis of DPM from acetate and CoA. The synthesis occurs in six steps (*i* - *vi*). CoA is consumed at reaction *i*, and regenerated at steps *ii-iv*. To prevent product inhibition and thermodynamically bias the system toward DPM formation, ADP (*vii*) and AMP (*vii* and *viii*) are recycled and pyrophosphate is hydrolysed (*ix*). **Panel B**. *The incorporation of acetate into DPM*. Acetate fragments are enzymatically concatenated to form the 6-carbon skeleton of DPM. Isotopic labels can be introduced at various points in the DPM synthesis to achieve a particular labeling outcome. The enyzmes used in the synthesis are as follows: *i*, acetyl-CoA synthetase; *ii, acetoacetyl-coA thiolase; iii*, hydroxymethylglutaryl-CoA synthase; *iv*, hydroxymethylglutaryl-CoA reductase; *v*, mevalonate kinase; *vi*, phosphomevalonate kinase; *vii*, pyruvate kinase; *viii*, adenylate kinase; *ix*, inorganic pyrophosphatase.

**Figure 2:**
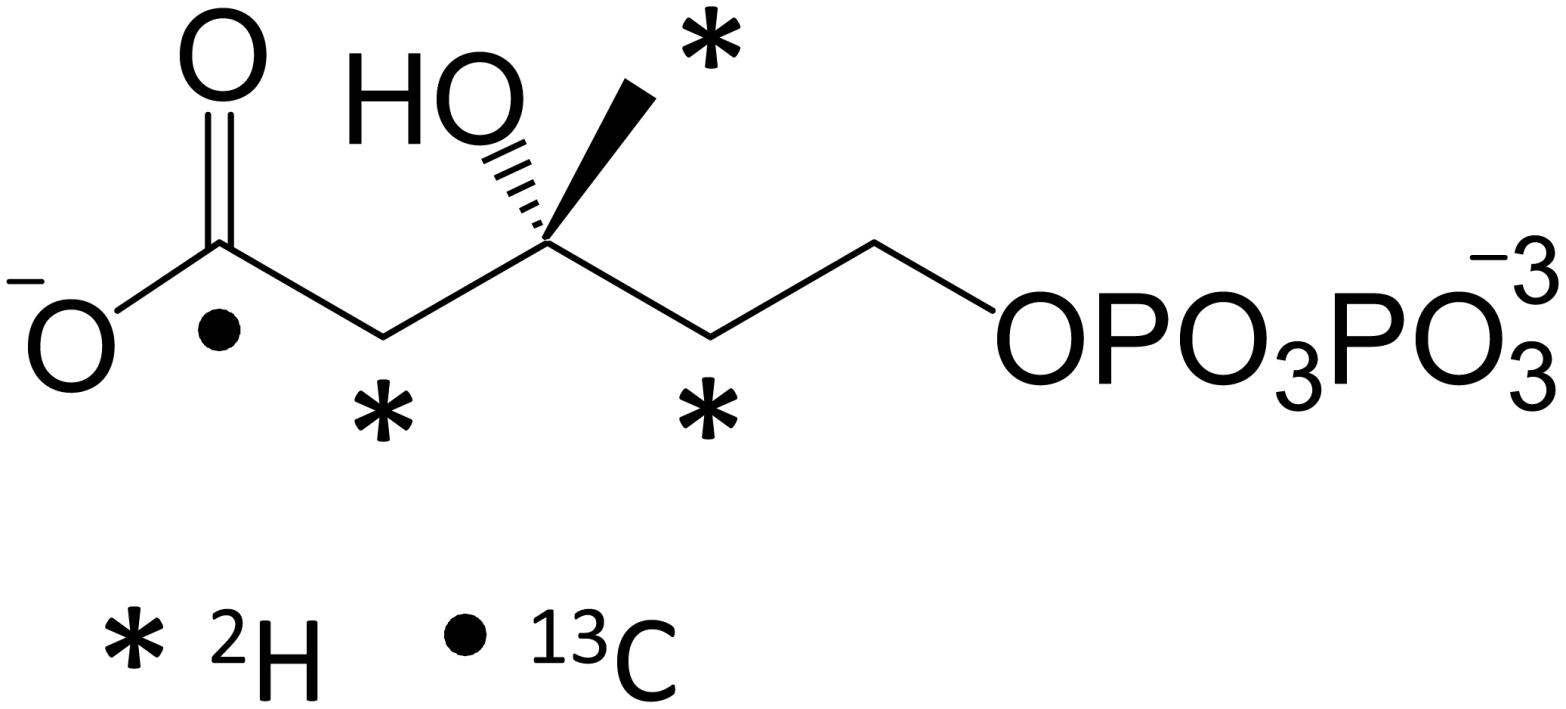
The isotopomers of (R)-diphosphomevalonate. Dots (•, [^13^C]) and asterisks (*, [^2^H]) mark the positions of heavy atoms in the synthesized compounds. Each mark represents a separate, singly-labeled compound. A triply-labeled compound, enriched at all of the [^2^H]-positions, was also synthesized.

The carbon backbones of isoprenoids are assembled from two fundamental building blocks, isopentenyl 5-pyrophosphate and dimethylallyl 5-pyrophosphate [37]–[40]. By linking the HMG-CoA reductase pathway, which produces mevalonate, to the mevalonate pathway, these building blocks can be enzymatically assembled from acetate, ATP, NAD(P)H, and CoA (Fig 1). Alternatively, they can be synthesized using the so-called non-mevalonate pathway [41], which is mechanistically more complex and less well defined [42]. Here, ten enzymes, including those that comprise the HMG-CoA reductase and mevalonate pathways [43]–[48] are strategically employed to accomplish efficient, high-yielding (>85%) single-pot syntheses of the intermediates and endproducts of the mevalonate pathway. Labeling strategies that regio-specifically position carbon and hydrogen isotopes into the building-block backbone are developed and used to synthesize and purify isotopomers of the immediate endproduct of the mevalonate pathway, diphosphomevalonate (DPM, Fig 2) [44]. Finally, the enzymatic system is shown to be robust and capable of producing pathway end-products in simple, aqueous solutions at levels that match or exceed the highest reported levels, which are only achieved using high-density fermentation.

## Materials and Methods

### Materials

Lactate dehydrogenase (rabbit muscle), pyruvate kinase (rabbit muscle), and inorganic pyrophosphatase (Baker's yeast) were purchased from Roche Applied Science. (*R, S*)-[^2^H_3_]methyl-mevalonolactone, (*R, S*)-mevalonolactone, acetyl-CoA, glutamate dehydrogenase (bovine liver), acetyl-CoA synthetase (Baker's yeast), myokinase (rabbit muscle) and lysozyme (bovine) were purchased from Sigma. Sodium acetate (^13^C, 99%), sodium acetate (^2^H, 99%) and D_2_O (99%) were purchased from Cambridge Isotope Laboratories, Inc. All other chemical reagents were of the highest grades available. Plasmids pET28efTR (encodes a bi-functional enzyme, *Enterococcus faecalis* acetoacetyl-CoA thiolase/HMG-CoA reductase), pET28efS2 A100G (encodes *Enterococcus faecalis* HMG-CoA synthase), and pET28-efR (encodes *Enterococcus faecalis* HMG-CoA reductase) were generous provided by Prof. V. W. Rodwell [43]. Mevalonate kinase (*Staphylococcus aureus*), phosphomevalonate kinase (*Streptococcus. pneumoniae*), and diphosphomevalonate decarboxylase (*Streptococcus. pneumoniae*) were expressed and purified as described previously [46], [49].

### Enzyme expression and purification

37°C LB/ampicillin media was inoculated with *E. coli* BL21(DE3) freshly transformed with the expression plasmid of interest. The cells were cultured to an OD_595_ of 0.8, protein expression was induced by the addition of isopropyl-1-thio-β-D-galactopyranoside (IPTG, 0.75 mM), and the incubation was continued for 4 h at 37°C. The culture temperature was then shifted to 18°C and incubation was continued for 16 h. The cells were then harvested by centrifugation (30 min, RCF 5,000 g, 4°C). The MVK [46], PMK [46] and DPM-DC (diphosphomevalonate decarboxylase) [46] expression vectors fuse a His_9_-GST-tag to the N-terminus of the enzyme; whereas, the acetoacetyl-CoA thiolase [47], HMG-CoA synthase [50] and HMG-CoA reductase [47] vectors fuse a His_6_-tag to the N-terminus. Dual-tag proteins were purified using a GST resin followed by a His resin. All buffers and columns were equilibrated at 4°C prior to use. Purification began by suspending cell pellets (5.0 ml/g cell paste) in **Buffer A** [H_2_KPO_4_ (50 mM), NaCl (140 mM), KCl (2.7 mM), pH 7.3] supplemented with lysozyme (0.10 mg/ml), PMSF (290 µM), and pepstatin A (1.5 µM). EDTA (1.0 mM) was added to Buffer A when purifying dual-tag systems. After suspension for 1 hr at 4°C, cells were disrupted by sonication and debris was removed by centrifugation (50 min, RCF 15,000 g, 4°C). Supernatants containing dual-tag proteins were loaded onto a Glutathione Sepharose 4 FF column equilibrated with Buffer A + EDTA, the column was then washed with three column volumes of the same buffer, and protein was eluted using Tris/Cl (250 mM, pH 8.0), KCl (500 mM) and reduced glutathione (10 mM). Supernatants containing singly tagged proteins, or GST-resin eluants containing dual-tag proteins, were loaded onto a Ni-NTA column equilibrated with **Buffer B** [H_2_KPO_4_ (50 mM), NaCl (300 mM), imidazole (10 mM), pH 8.0]. The column was washed with **Buffer C** [H_2_KPO_4_ (50 mM), NaCl (300 mM), imidazole (20 mM), pH 8.0], and fusion protein was eluded with **Buffer D** [H_2_KPO_4_ (50 mM), NaCl (300 mM), imidazole (300 mM), β-mercaptoethanol (β-ME) (10 mM), pH 8.0]. Glycerol was then added to the singly-tagged eluants (5% v/v) and stored (see below). Tags were removed from the dual-tag proteins by incubation with PreScission protease [51] during overnight dialysis at 4°C against Hepes/K^+^ (50 mM, pH 8.0) containing DTT (10 mM, dithiothreitol) and KCl (100 mM). Following proteolysis, the dialysate was passed over a GSTrap column to remove the GST-tagged protease. The purity of the single- and double-tags proteins was estimated, using SDS-PAGE, at >85 and >95%, respectively. Eluants containing purified proteins were frozen rapidly and stored at −80°C.

### Enzymatic assays

To establish conditions for the synthesis of DPM, the activity of each enzyme was assessed under the synthesis conditions. Apparent kinetic constants were extracted from reaction progress curves [11] and were in good agreement with published values (Table 1). *Acetyl-CoA synthetase activity* was monitored by coupling the production of AMP to the oxidation of NADH [52]. The assay conditions were as follows: inorganic pyrophosphatase (4.0 U/ml), myokinase (4.0 U/ml), PK (4.0 U/ml), LDH (lactate dehydrogenase, 8.0 U/ml), NADH (3.0 mM, ε_398_ = 0.136 mM^−1^ cm^−1^), acetate (2.0 mM), CoA (2.0 mM), ATP (4.0 mM), PEP (6.0 mM), MgCl_2_ (1.0 mM + [nucleotide]), KCl (50 mM), β-ME (10 mM). *Acac-Coa thiolase activity* was monitored by following the appearance of acac-CoA (acetoacetyl-CoA) at 302 nm [53]. The conditions were: Ac-CoA (acetyl-CoA) (6.0 mM), MgCl_2_ (2.0 mM). *HMG-CoA synthase activity* was monitored at 386 nm (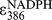 = 0.61 mM^−1^ cm^−1^) by coupling the production of 3-hydroxy-3-methyl glutaryl-CoA to the oxidation of NADPH using HMG-CoA reductase. The conditions were: HMG-CoA reductase (1.0 µM), acac-CoA (1.0 mM), Ac-CoA (1.0 mM), NADPH (1.5 mM), KCl (50 mM), β-ME (10 mM). *HMG-CoA reductase activity* was monitored by following oxidation of NADPH. The conditions were: 3-hydroxy-3-methyl glutaryl-CoA (0.50 mM), NADPH (0.20 mM), KCl (50 mM), β-ME (10 mM). *Mevalonate kinase activity* was monitored by coupling the production of ADP to the oxidation of NADH [46], [49]. The conditions were: PK (4.0 U/ml), LDH (8.0 U/ml), NADH (200 µM, ε_339_ = 6.22 mM^−1^ cm^−1^), PEP (7.0 mM), mevalonate (135 µM), ATP (5.0 mM), MgCl_2_ (1.0 mM + [nucleotide]), KCl (50 mM), β-ME (10 mM). *Phosphomevalonate kinase activity* was monitored by coupling the production of ADP to the oxidation of NADH [46], [49]. The conditions were identical to those used for mevalonate kinase except phosphomevalonate (50 µM) replaced mevalonate. *DPM Decarboxylase activity* was monitored by coupling the production of ADP to the oxidation of NADH [46], [49]. The conditions were identical to those used for mevalonate kinase except diphosphomevalonate (50 µM) replaced mevalonate. In all cases, reactions were buffered with Hepes/K^+^ (50 mM), pH 8.0, and T = 25±2°C.

**Table 1:** Enzymes used in the synthesis of DPM.

aEnzyme	EC #	Gene	Source	Substrate	d *K*_m_ (mM)	d *k*_cat_ (sec^−1^)
bACS	6.2.1.1	*Acs1*	*S. cerevisiae*	Acetate CoA	0.28 0.24	10
cACT	2.3.1.9	*mvaE*	*E. faecalis*	Ac-CoA	0.60	2.3
cHMGS2	2.3.3.10	*mvaS*	*E. faecalis*	acac-CoA Ac-CoA	0.015 0.35	1.0
cThRed	1.1.1.34	*mvaE*	*E. faecalis*	HMG-CoA	0.023	0.55
cHMGR	1.1.1.34	*mvaE*	*E. faecalis*	HMG-CoA	0.020	0.67
cMVK	2.7.1.36	*mvaK1*	*S. aureus*	Mev	0.027	19
cPMK	2.7.4.2	*mvaK2*	*S. pneumoniae*	P-mev	0.0042	5.0
bPK	2.7.1.40	*PKM2*	*O. cuniculus*	PEP	0.040	160
bMK	2.7.4.3	*AK1*	*O. cuniculus*	AMP	e0.50	e410
bPP_i_ase	3.6.1.1	*Ppa1*	*S. cerevisiae*	PP_i_	e0.0050	e260

a Abbreviations: ACS, acetyl-CoA synthetase; ACT, acetoacetyl-CoA thiolase; HMGS2, HMG-CoA synthase; ThRed, acetoacetyl-CoA thiolase/HMG-CoA reductase (dual-function enzyme); HMGR, HMG-CoA reductase; MVK, mevalonate kinase; PMK, phosphomevalonate kinase; PK, pyruvate kinase; MK, myokinase; PP_i_ase, inorganic pyrophosphatase.

b Obtained from commercial sources.

c Expressed in *E. coli* and purified.

d Standard errors are <5% in all cases (see *Materials and Methods*).

e Parameters taken from literature (MK [77], [78], PP_i_ase [79], [80]).

### The synthesis of (*R*)-diphosphomevalonate

DPM was synthesized in a one-pot reaction using the following conditions: Ac-CoA synthetase (2.0 µM), acac-CoA thiolase (2.0 µM), HMG-CoA synthase (4.0 µM), HMG-CoA reductase (2.0 µM), mevalonate kinase (2.0 µM), phosphomevalonate kinase (3.0 µM), pyruvate kinase (5.0 U/mL), myokinase (2.0 U/ml), inorganic pyrophosphatase (2.0 U/ml), ATP (5.0 mM), PEP (10 mM), acetate (12 mM), CoA (5.0 mM), NADPH (10 mM), KCl (50 mM), MgCl_2_ (1.0 mM + [ATP]), β-ME (10 mM), Hepes/K^+^ (50 mM), pH 8.0, T = 25±2°C. Reactions progress was monitored by following the oxidation of NADPH associated with the HMG-CoA reductase reaction. DPM formation was assayed by adding an aliquot the DPM-synthesis reaction into a DPM decarboxylase assay mixture (DPM-DC (0.10 µM), PK (4.0 U/ml), LDH (8.0 U/ml), NADH (200 µM), PEP (4.0 mM) ATP (2.0 mM), MgCl_2_ (1.0 mM + [nucleotide]), KCl (50 mM), β-ME (10 mM), Hepes/K^+^ (50 mM), pH 8.0, T = 25±2°C) and monitoring NADH oxidation at 340 nm. The assay-reaction dilution was sufficient (330-fold dilution) to prevent the HMG-CoA reductase reaction from contributing significantly to the measurement. The reactions yielded essentially quantitative conversion of acetate to the endproduct, DPM.

### The synthesis of labeled acetyl-CoA precursors

The synthesis of regiospecifically labeled DPM requires appropriately labeled Ac-CoA. Labeled Ac-CoA precursors were synthesized using the following conditions: acetyl-CoA synthetase (2.0 µM), pyrophosphatase (2.0 U/ml), labeled acetate (4.0 mM), CoA (4.0 mM), ATP (4.0 mM), MgCl_2_ (5.0 mM), Hepes/K^+^ (50 mM), pH 8.0. The reactants were mixed gently for 10 hr at T = 25±2°C. Reaction progress was monitored by assaying aliquots of the reaction for AMP synthesis using the Ac-CoA synthetase assay described above. The conversion of CoA to labeled Ac-CoA was >95%.

### Synthesis of acac-CoA

The synthesis of acac-CoA was achieved using the conditions identical to those described for the synthesis of Ac-CoA with the exception that acac-CoA thiolyase (2.0 µM) and DTNB (10 mM, 5, 5'-Dithio-bis(2-nitrobenzoic acid) were present. DNTB reacts with CoA and was used to draw the acac-thiolase reaction forward. The DTNB reaction was monitored at 412 nM [54]. Acac-CoA formation was monitored at 302 nm (*see*, *Enzymatic assays*, *Materials and Methods*). The reaction reached completion after approximately 17 hr, after which >98% acetyl-CoA had converted to acac-CoA. The reaction was filtered (10 kDa membrane) to remove enzymes prior to using the acac-CoA in subsequent syntheses.

### The synthesis of [1-^13^C]DPM or [2-^2^H_2_]DPM

Labeled Ac-CoA (^13^C or ^2^H) was prepared from CoA and labeled acetate as described above (see, *Synthesis of labeled acetyl-CoA precursors*). Labeled DPM was synthesized by adding the following reagents to the labeled Ac-CoA reaction mixture: PK (10 U/mL) (U, µmoles product formed min^−1^ at a saturating substrate), HMG-CoA synthase (4.0 µM), HMG-CoA reductase (2.0 µM), MVK (2.0 µM), PMK (1.0 µM), PEP (10 mM), NADPH (5.0 mM), unlabelled acac-CoA (2.0 mM), ATP (5.0 mM), KCl (50 mM), and β-ME (10 mM). The unlabeled acac-CoA was prepared as describe above (see, *Synthesis of acac-CoA*). The reaction was stirred gently overnight (∼16 h, 25±2°C), at which point >97% of the labeled Ac-CoA had incorporated into DPM. The quantitation of DPM is described above (see, *The synthesis of (R)-diphosphomevalonate*).

### The synthesis of DPM from acetate at high concentration

DPM synthesis was accomplished in a one-pot reaction using the following conditions: Ac-CoA synthetase (5.0 µM), acac-CoA thiolase (7.0 µM), HMG-CoA synthase (10 µM), HMG-CoA reductase (7.0 µM), mevalonate kinase (5.0 µM), phosphomevalonate kinase (3.0 µM), pyruvate kinase (10 U/mL), myokinase (5.0 U/ml), inorganic pyrophosphatase (5.0 U/ml), ATP (100 mM), PEP (800 mM), acetate (340 mM), CoA (5.0 mM), NADPH (300 mM), MgCl_2_ (110 mM), β-ME (10 mM), Hepes/K^+^ (50 mM), pH 8.0, T = 25±2°C. Reaction progress was monitored as described above (see, Enzymatic Assays). Under the high ionic strength conditions of this reaction, the conversion of acetate to DPM decreased to sixty-three percent.

### The synthesis of IPP from acetate at high concentration

IPP synthesis was accomplished in a one-pot reaction using the following conditions: Ac-CoA synthetase (7.0 µM), acac-CoA thiolase (10 µM), HMG-CoA synthase (12 µM), HMG-CoA reductase (10 µM), mevalonate kinase (7.0 µM), phosphomevalonate kinase (5.0 µM), diphosphomevalonate decarboxylase (3.5 µM), pyruvate kinase (20 U/mL), myokinase (7.0 /ml), inorganic pyrophosphatase (7.0 U/ml), ATP (200 mM), PEP (800 mM), acetate (340 mM), CoA (5.0 mM), NADPH (300 mM), MgCl_2_ (220 mM), β-ME (10 mM), Hepes/K^+^ (50 mM), pH 8.0, T = 25±2°C. The conversion of acetate to IPP yields nine IPP-equivalents of pyruvate (two equivalents for each of the three Ac-CoAs required to synthesize HMG-CoA (hydroxymethylglutaryl-CoA), two for conversion of Mev to DPM and one for the decarboxylation of DPM to IPP). Pyruvate was quantitated by adding an aliquot of the IPP-synthesis reaction to a lactate dehydrogenase assay mixture: (LDH (8.0 U/ml), NADH (200 µM), KCl (50 mM), β-ME (10 mM), Hepes/K^+^ (50 mM), pH 8.0, T = 25±2°C). Dilution of the synthesis reaction was sufficient (>500-fold) to prevent enzymes from the reaction from contributing significantly to the pyruvate measurements. Sixty-nine percent of the acetate was converted to DPM.

### The synthesis of DPM from (R/S)-mevalonate at high concentration

DPM synthesis was accomplished in a reaction using the following conditions: mevalonate kinase (5.0 µM), phosphomevalonate kinase (3.0 µM), pyruvate kinase (10 U/mL), (*R/S*)-mevalonate (370 mM), ATP (50 mM), PEP (350 mM), MgCl_2_ (60 mM), β-ME (7.0 mM), Hepes/K^+^ (50 mM), pH 8.0, T = 25±2°C. Reaction progress was monitored as described above (see, Enzymatic Assays). It should be noted that mevalonate kinase converts only the *R*-isomer of mevaloante to phosphomevalonate [55], and the enantiomeric composition of commercial (*R/S*)-mevalonate is 1∶1 [11]; hence, a maximum of 50% of the commercial product can be converted to DPM. Seventy-one percent of the (*R*)-mevalonate in the (*R/S*)-mixture was converted to DPM.

### The synthesis of IPP from (R/S)-mevalonate at high concentration

IPP synthesis was accomplished in a reaction using the following conditions: mevalonate kinase (5.0 µM), phosphomevalonate kinase (3.0 µM), diphosphomevalonate decarboxylase (1.6 µM), pyruvate kinase (10 U/mL), (*R/S*)-mevalonate (375 mM), ATP (50 mM), PEP (450 mM), MgCl_2_ (60 mM), β-ME (7.0 mM), Hepes/K^+^ (50 mM), pH 8.0, T = 25±2°C. The conversion of (*R/S*)-mevalonate to IPP was monitored by following the formation of pyruvate using lactate dehydrogenase (see, *The synthesis of IPP from acetate at high concentration*). Seventy-seven percent of the (*R*)-mevalonate in the (*R/S*)-mixture was converted to IPP.

### The purification (*R*)-diphosphomevalonate

To maximize the purity and recovery of DPM, PEP (which chromatographs near DPM) was converted to pyruvate by adding one PEP-equivalent of ADP to the synthesis reaction mixture. Small and large molecules were separated by ultrafilitration (10-kDa cutoff). The small-molecule filtrate was passed through a 35 mL bed of anion exchange resin (AG MP-1) equilibrated with Hepes/K^+^ (10 mM, pH 7.5), and the column was “washed” with five volumes of equilibration buffer. The compounds were eluted using a 750 ml, linear salt gradient (0–1.0 M KCl) at 2.0 mL/min. DPM eluted at 0.32 mM KCl and contained <1% nucleotide. To remove excess KCl and concentrate the DPM, the purified compound was loaded onto a 5.0 ml bed of AG MP-1 equilibrated with NH_4_HCO_3_ (10 mM, pH 7.5). The column was then “washed” with five volumes of NH_4_HCO_3_ (10 mM, pH 7.5) before eluting the DPM with 1.8 volumes of NH_4_HCO_3_ (350 mM, pH 7.5). Excess NH_4_HCO_3_ was removed by rotary evaporation at 45°C. The desalted compounds were dissolved in ultra pure water (2.0 mL) and the solution was adjusted to pH 7.5 with KOH. NH_4_HCO_3_ in the desalted, purified DPM was measure using an enzymatic assay that couples the reduction of NADP^+^ to the synthesis of glutamate from 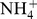 and α-ketoglutarate [56]. The assay conditions were as follows: α-ketoglutarate (5.0 mM), NADP^+^ (0.20 mM), glutamate dehydrogenase (14 U/mL), Hepes/K^+^ (45 mM) pH 8.0 at T = 25±2°C. The 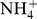/DPM stoichiometry was ∼4∶1. The DPM concentration and purity, presence of mevalonate and phosphomevalonate, were determined spectrophotometrically using the assay described above (see, *Enzymatic assay*), and the purified compounds were stored in Hepes/K^+^ (10 mM, pH 8.0) at −80°C.

### NMR protocols

One dimensional NMR was used to confirm the structure and isotopic labeling of the DPM isotopomers. A Bruker DRX 300 MHz spectrometer equipped with a 5 mm broadband probe was used to acquire data. Sample temperature was 25±2°C. Proton spectra were the average of 32 scans (64K points each) acquired over 20 ppm using a 1.0 s recycle delay. The residual water signal was suppressed by presaturation of the HOD resonance. Spectra were processed with 1.0 Hz line broadening, and proton chemical shifts were referenced to 3-(trimethylsilyl) propionate [57]. Proton-decoupled carbon spectra were the average of 100 scans (61K points each) acquired over 315 ppm using a 3.0 s recycle delay. Spectra were processed with a 1.5 Hz line broadening, and chemical shifts were referenced indirectly [57]. Proton-decoupled phosphorus spectra were the average of 256 scans (64K points each) acquired over 50 ppm using a 6.0 s recycle delay. Spectra were processed with a 3.0 Hz line broadening, and chemical shifts were referenced to phosphocreatine [58].

## Results and Discussion

### The enzymatic synthesis of DPM

Diphosphomevalonate is synthesized from acetate, ATP and NADPH in six consecutive enzymatic steps (*i* - *vi*, Fig 1A). The first four reactions produce mevalonate from 3 acetate, 3 ATP, and 2 NADPH [55], [59], [60]. CoA, which acts as an acetyl-carrier, is consumed in reaction *i*, and regenerated in reactions *ii*, *iii* and *iv* (see, dashed green arrows, Fig 1A). Reactions *v* and *vi* are catalyzed by kinases that phosphorylate mevalonate to produce the pyrophosphoryl-group of DPM. To bias the reactions toward the endproduct and avoid product inhibition, ADP and AMP were recycled to ATP using pyruvate kinase and myokinase, and PP_i_ was hydrolyzed to P_i_ using inorganic pyrophosphatase. In total, nine enzymes were used in the synthesis [43]–[48].

Enzymes *ii* - *vi* were cloned, expressed in *E. coli* and purified (see, *Materials and Methods*); *i* and *vii - ix* were obtained from commercial sources. The purified enzymes were 80–95% pure, as judged by Comassie staining [61] of SDS PAGE [62] gels, and were obtained in yields of 30–40 mg pure protein/liter of *E. coli*. The kinetic constants of the purified enzymes were determined under the conditions used for the synthesis, and were in good agreement with literature values (Table 1). The assays are described in *Enzymatic Assays* (see, *Materials and Methods*). The enzymes showed no significant loss of activity over an 8 month period when frozen rapidly and stored at −80°C in Hepes (50 mM, pH 8.0), 150 mM KCl, 5% glycerol (v/v).

The relative enzyme concentrations used in the DPM syntheses were determined empirically by adjusting concentrations such that flux through the pathway was not rate-limited by any single step. This was accomplished by setting PMK (*vi*) at a fixed concentration and titrating each preceding enzyme successively until the DPM-synthesis rate was 80–90% of the maximum rate achievable at each step. For example, MVK (*v*) was titrated at a fixed concentration of PMK until the rate of DPM synthesis became independent of MVK concentration – the maximum rate. The MVK concentration was then adjusted to allow 80–90% of the maximum rate, and an analogous procedure was performed with HMG-CoA reductase (*iv*). The procedure was performed in succession for each enzyme in the pathway to determine the relative enzyme concentrations to be used in the synthesis. Once relative concentrations were established, the absolute concentrations were set to achieve the desired reaction times, which ranged from 8–72 hr. Mevalonate kinase from *S. aureus* was selected because, unlike the *S. pneumoniae* enzyme, it is not allosterically inhibited by DPM [11].

Substrates were set at saturating, sub-inhibiting concentrations. ATP, a substrate for five of the enzymes (*i*, *v*, *vi*, *vii* and *viii*), was set at 5.0 mM, which ranges from 5.8–68×*K*_m_. Typical substrate concentrations of the other reactants were as follows: acetate (12 mM, 42×*K*_m_); CoA (2.0 mM , 8.0×*K*_m_); NADPH (10 mM, 320×*K*_m_); and PEP (10 mM, 250×*K*_m_). Under these conditions, and using the enzyme concentrations detailed in *Synthesis of (R)-diphosphomevalonate* (*Materials and Methods*), ∼98% of the acetate was incorporated into DPM in this single-pot reaction.

### The incorporation of isotopes into DPM

The regiospecific incorporation of isotopes has proven extremely valuable in the elucidation of metabolism [63]–[65] and determining enzyme mechanism [66]. Indeed, this was the basis for the discovery of the non-mevalonate pathway of isoprenoid biosynthesis [40]. The enzymatic scheme shown in Fig. 1 offers a flexible and efficient means of synthesizing numerous radiolabeled and stable isotopomers of mevalonate, many of which are not commercially available. The six-carbon backbone of DPM is constructed in the first three enzymatic steps of the scheme (*i* – *iii*). Each step adds a single acetate to the CoA thioester R-group. The pattern of acetate incorporation into the R-group, and ultimately DPM, is shown in Figure 1B. Acetate is first esterfied onto the CoA thiol, and subsequent two-carbon units are added by forming carbon-carbon bonds with the existing R-group. Isotopes can be incorporated into specific positions in DPM (Fig 2) using labeled acetate or acetyl-CoA, or *via* solvent exchange with exchange-sensitive intermediates. Achieving certain labeling patterns required removal of enzymes by ultrafiltration at intermediate stages of the synthesis, and/or that reactions were run in D_2_O (see below).

### The synthesis of [2, 4, 6,-^2^H_7_]- and [6-^2^H_3_]DPM

The compounds were synthesized in approximately 50 mg quantites in one-pot reactions using commercial [2-^2^H_3_]acetate or (*R, S*)-[6-^2^H_3_]mevalonolactone as starting material (see, *Supplementary Material*). Reactions were complete after 22 hrs, and virtually quantitative conversion of starting material to DPM was achieved in all cases. Approximately ∼86% of the maximum theoretical maximum yield of DPM was obtained after purification (see, *Material and Methods*). The labeling of DPM was confirmed using ^1^H NMR (Fig. S1).

### Synthesis of [4-^2^H_2_]DPM

**Figure 3:**
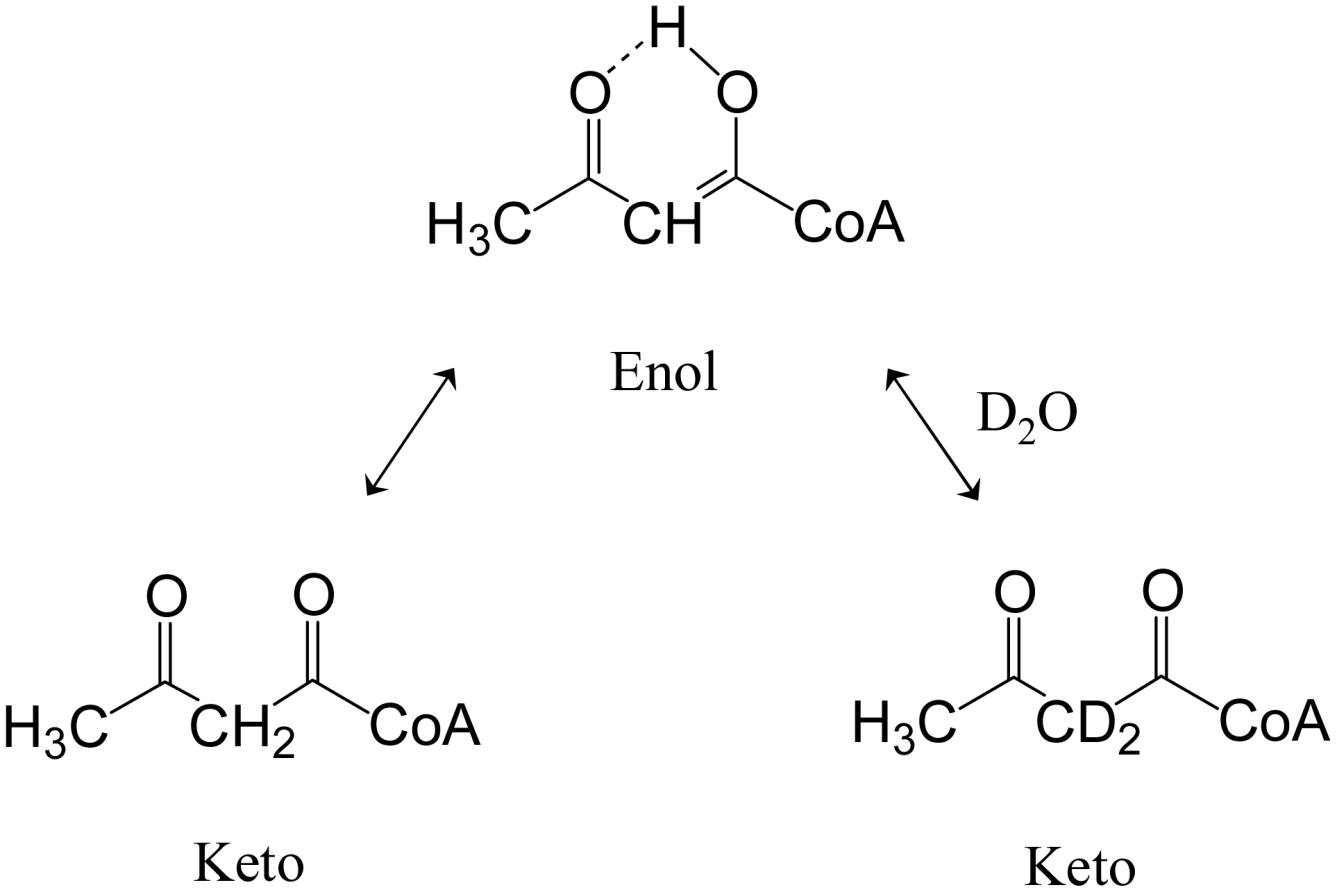
Tautomerization of acetoacetyl-CoA. Tautomerization allows H/^2^H-exchange at the C_2_-position of the 3-oxobutyryl-moiety of acac-CoA, and thus provides the precursor for the synthesis of [4-^2^H_2_]DPM.

The synthesis of [4-^2^H_2_]DPM was carried out in several steps. First, unlabeled Ac-CoA was synthesized using acetyl-CoA synthetase (*i*) and inorganic pyrophosphatase (*ix*) (see, *Synthesis of acetyl-CoA*, *Materials and Method*). Acetyl-CoA thiolyase (*ii*) and DTNB (in excess over Ac-CoA) were then added to form acac-CoA. DTNB reacts quantitatively with CoA [54] and was used to draw the unfavorable acac-CoA-forming reaction to completion [47]. Acac-CoA tautomerizes [67], and its enol-form exchanges protons with solvent (Fig. 3). To streamline the synthesis, both enzymatic reactions were run in D_2_O. ^1^H NMR confirmed that exchange was complete and occurred exclusively at the C_4_-position of DPM (Fig. S1). It is notable that this exchange suggests the possibility of using equilibrium isotope exchange to produce Ac-CoA in which the methyl-protons have been exchanged with solvent. To attach the third acetate without forming unlabelled acac-CoA, which would dilute isotopic enrichment, the Ac-CoA thiolyase was removed by ultrafiltration before adding the reactants that complete the synthesis of DPM (see, *Synthesis of [4-^2^H_2_]-DPM, Supplementary Material*). The reactions were essentially quantitative and the production of DPM was ∼96% of the theoretical maximum.

### The synthesis of [1-^13^C]- and [2-^2^H_2_]DPM

The strategy used to synthesize these compounds was similar to that used in the synthesis of [4-^2^H_2_]DPM. Unlabeled acac-CoA was synthesized, the enzymes used in the synthesis were “strained” from the reaction by ultrafiltration, labeled Ac-CoA was then added along with the enzymes, and reagents needed to complete the synthesis of DPM (see, *Materials and Methods*). Care was taken to ensure that the Ac-CoA remaining in the acac-CoA synthesis reaction was ∼0.2% of the labeled Ac-CoA used in the subsequent conversion to DPM. The yield was quantitative; DPM levels reached ∼95% of the theoretical maximum.

### Confirming the structure and labeling patterns of the compounds

**Figure 4:**
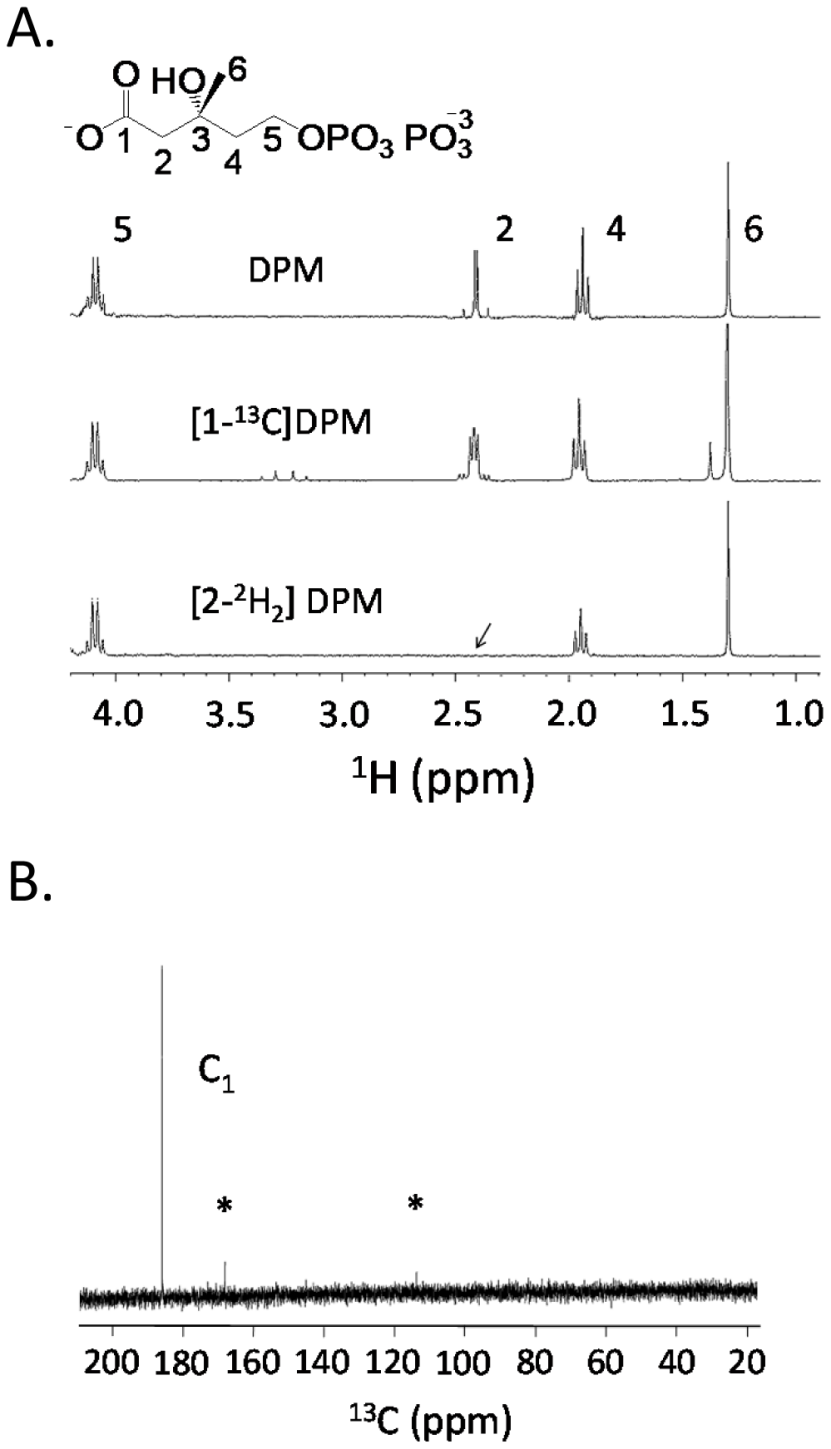
^1^H and ^13^C NMR spectra of (R)-diphosphomevalonate isotopomers. Spectra indicate specificity and efficiency of labeling. **Panel A**. ^1^H NMR spectra of labeled and unlabeled DPM. The efficiency and specificity of [^2^H_2_]-labeling were estimated at >98% and >95%, respectively. **Panel B**. ^13^C NMR spectrum of [1-^13^C]DPM. The resonance at 181 ppm corresponds to C_1_. The efficiency of labeling at C_1_ is estimated at >92% (see *Results and Discussion*). Based on the absence of non-C_1_ signals and the S/N, the labeling specificity is calculated at >98%. Asterisks indicate instrumental artifacts.

The specificity and efficiency of isotopic labeling were assessed using ^1^H and ^13^C NMR. Deuterium incorporation at a given position was assessed by quantitating the loss of proton signal at that position. The ^1^H NMR spectra of the synthesized compounds are compiled in Figures 4A and S1. In all cases, proton signal at the targeted position(s) was below detection (i.e., >97% incorporation efficiency) and the integrated intensities of the remaining proton peaks were identical within error (±3%); thus, deuterium did not incorporate significantly into positions other than the target sites. Comparison of the ^1^H spectra of [1-^13^C]- and natural abundance C_1_-DPM reveals that the AB quartet associated with the C_2_ of DPM (2.41 ppm) is split to into an ABX pattern by the incorporation of ^13^C (Fig 4A). This splitting is consistent only with ^13^C incorporation at C_1_. If the synthesis had resulted in a significant fraction of natural abundance C_1_-DPM, the AB and ABX resonances are expected to overlap. Close inspection of the upfield doublet of the ABX pattern gives no indication of the AB species (Fig 4 inset) indicating that the incorporation efficiency is quite high (>95%). The labeling specificity of [1-^13^C]DPM is given by the ^13^C spectrum (Fig 4B), which shows the expected C_1_-resonance [68] and no detectible signal at the positions associated with the other carbon atoms in the molecule (dotted arrows). The integrity of the pyrophosphoryl moiety was confirmed using ^31^P NMR (Figure S2).

### The synthesis of highly concentrated DPM and isopentenyldiphosphate

**Figure 5:**
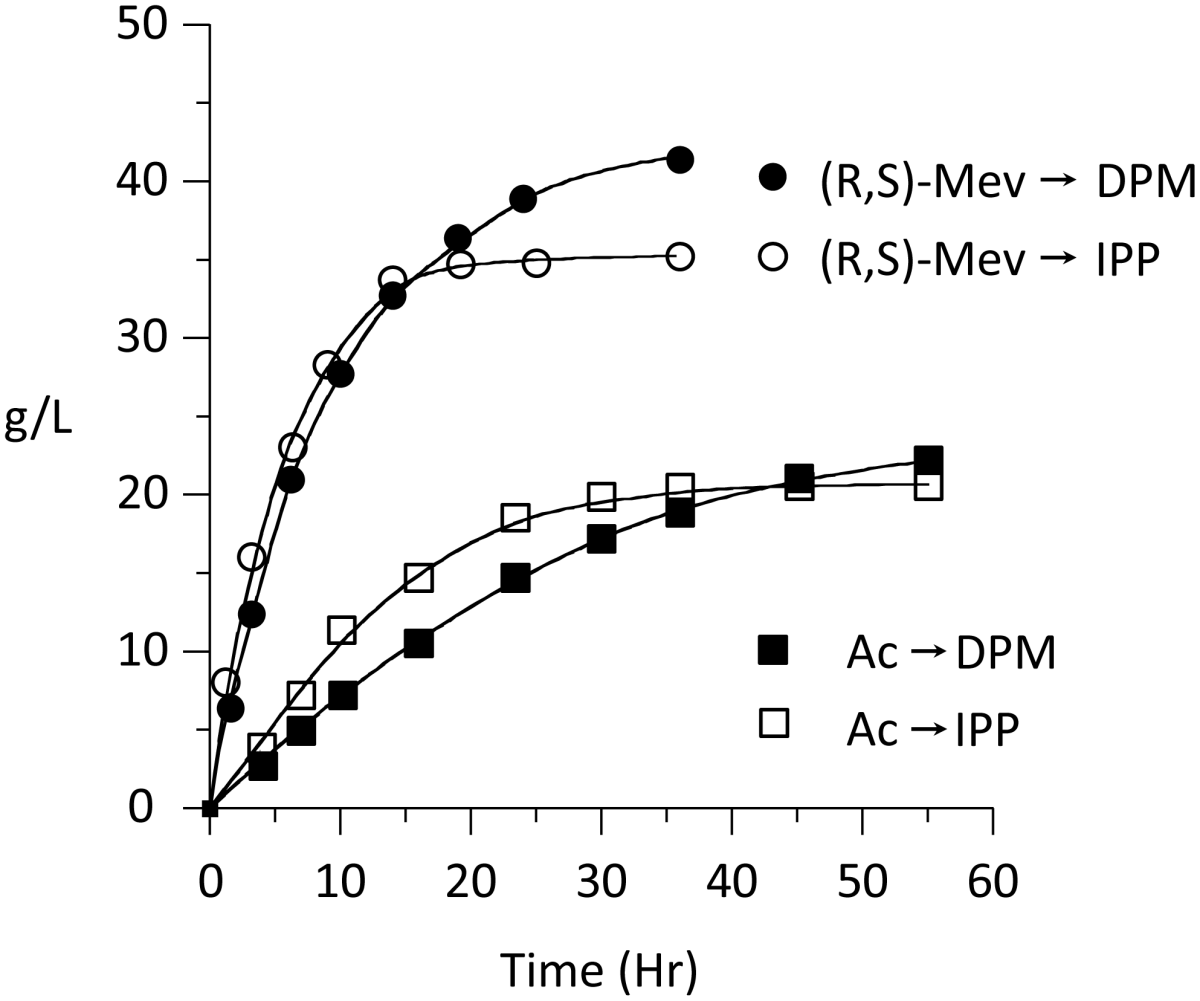
DPM and IPP synthesis at high reactant concentration. DPM and IPP were synthesized in separate, single-pot reactions. Reactions were initiated from acetate, or (R,S)-mevalonate. The conditions are described in *Materials and Methods*. Reactions initiated with acetate yielded 69% conversion of acetate to IPP (○), or 63% conversion of acetate to DPM (•). Reactions starting with (R,S)-mevalonate yielded 77% conversion of (*R*)-mevalonate to IPP (□), or 73% conversion of (R)-mevalonate to DPM (▪). The data points represent the average of results from three independent experiments.

Given the considerable societal value of isoprenoids, the difficulties obtaining them, and the current efforts to bio-synthesize these compounds at commercial scale, it was of interest to assess the potential of the *in-situ* enzymatic synthesis to produce large quantities of product. Toward this end, the velocity of the acetate-to-DPM conversion was studied as a function of initial-reactant concentration with the goal of determining the highest, useful concentrations. The system proved remarkably robust. Only slight inhibition (∼30%) was observed at 0.50 M acetate. PEP and NADPH could be increased to near saturation (∼500 and 200 mM, respectively) without significant decrease in velocity, and ATP could be added to 0.15 M without inhibition or noticeable precipitation. The concentration-optimized system contained acetate, ATP, PEP and NADPH at 0.35, 0.10, 0.40, 0.30 M, respectively, and yielded DPM and IPP at 22 and 18 g/liter, respectively −63% and 69% conversions of acetate to product (Fig 5). Product formation was limited by the solubility of nucleotide and high ionic strength of these reactions.. To assess whether the enzymatic system was capable of producing even higher product concentrations, DPM and IPP synthesis was initiated from mevalonate. Reactions contained (*R/S*) mevalonate, ATP, and PEP at 0.370, 0.05, 0.35 M, respectively, and yielded DPM and IPP at 42 and 35 g/liter, respectively −73% and 76% product yields (Fig 5). The reactions conditions are further described in *Materials and Methods* (see, *Reactions that yield highly concentrated product*).

The syntheses outlined in the preceding paragraph are highly scalable. Reaction yields were independent of volume from 0.10 ml–1 liter and are expected to be similar at larger volumes. Under the high ionic strength conditions of these assays, the enzymes proved to be quite stable. The majority lost ≤20% of their activity over 2 days at room temperature. The exceptions were acetoacetyl-CoA thiolyase and inorganic pyrophosphatase, which lost 63% and 72% of their activity, respectively, over this time period.

Attempts to genetically engineer plants and bacteria to produce commercial quantities of isoprenoids have met with variable success. Artemisinin, a potent antimalarial, is currently isolated from the *quinghao* plant (*Artemisia annua*) at ∼3 mg/g dry weight. In contrast, genetically engineered tobacco produces artemisinin at ∼0.8 mg/g dry weight [69], and transgenic yeast secrete artemisinic acid (an artemisinin precursor [70] at 100 mg/liter [71]. Using *E. coli* as the host, pathway optimization has yielded ∼0.3 g/liter of artemisinic acid in shaking flasks [34], [72], high-density batch fermentation of engineered *E. coli* has produced yields as high as 23g/liter [73] and recent breakthroughs in understanding of the pathway have produced artemisinic acid at ∼25 g/liter in moderately high-density *E. coli* cultures [74]. Similar efforts in *E. coli* have produce taxadiene (a precursor of Taxol, an anticancer therapeutic) at ∼1g/liter in shaking flasks [75]. Production of farnesol, a relatively simple isoprenoid and potential biofuel [29], has reached 130 mg/liter in engineered *E. coli* grown in shaker flasks [76].

While these efforts have helped define the complexities associated with expressing and controlling the isoprenoid biosynthetic pathway in living organisms, only fermentation in conjunction with genetic engineering is yielding product quantities required for successful commercial application. The cell-free approach described here yields product quantities that are comparable to, or exceed those achieved in high-density fermentation and have the advantage that product is formed in a simple aqueous system from which it can be recovered readily.

## Conclusions

The enzymes that comprise the HMG CoA reductase and mevalonate pathways have been used along with enzymatic substrate-recycling and product-removal systems to efficiently synthesize intermediates and end products of these pathways in high yield. Strategies for using these enzymes to regio-specifically position isotopes in the isoprenoid backbone are described and used to synthesize and purify isotopomers of DPM, the immediate end product of the mevalonate pathway. The enzymatic platform is robust and produces isoprenoid precursors, DPM and IPP, in quantites ranging from 20–40 g/liter. These values meet or exceed all published values for isoprenoid production using genetically engineered organisms. The platform produces product in simple aqueous solutions, which, in most cases, will make isoprenoid isolation far simpler than extraction from high-density fermentums. These favorable attributes recommend the enzymatic platform as a valuable alternative to cell-culturing methods as a clean, sustainable, non-biocompetitive method for the production of isoprenoids.

## Supporting Information

Figure S1 **^1^H NMR spectra of DPM isotopomers.** The specificity and efficiency of labeling of the isotopomers were estimated based on the integration of the ^1^H signals. The results were as follows: [4-^2^H_2_]DPM (96%, 95%), [6-^2^H_3_]DPM (97%, 95%) and [2, 4, 6-^2^H_7_]DPM (96%, 97%).
(TIF)

Figure S2 **^31^P NMR spectrum of DPM.** The resonance positions, splitting pattern and nearly identical integrated intensities of the <- and -resonances indicate an intact pyrophosphoryl-moiety. The asterisk identifies the resonance of phosphocreatine, which was added as an internal standard. The absence of a peak at ∼5 ppm indicates that phophomevalonate is undetectable.
(TIF)

Text S1 (DOCX)
