# Supplementary figures and images for: An Enzymatic Platform for the Synthesis of Isoprenoid Precursors
Source: PLoS One. 2014 Aug 25;9(8):e105594. doi: 10.1371/journal.pone.0105594 (PMC4143292; doi:10.1371/journal.pone.0105594)

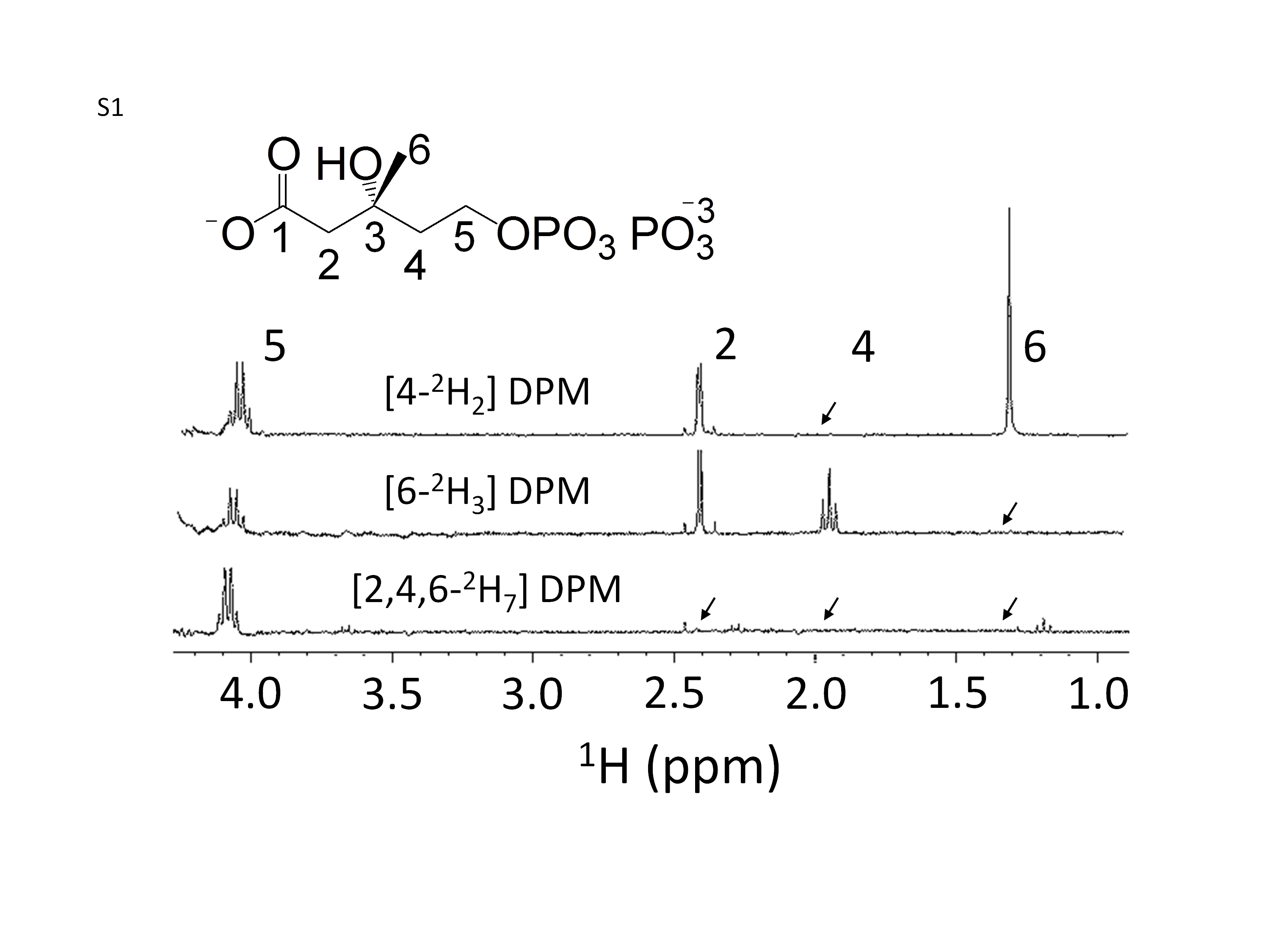

Supplement: Figure S1 — 1H NMR spectra of DPM isotopomers. The specificity and efficiency of labeling of the isotopomers were estimated based on the integration of the 1H signals. The results were as follows: [4-2H2]DPM (96%, 95%), [6-2H3]DPM (97%, 95%) and [2, 4, 6-2H7]DPM (96%, 97%). (TIF) [file pone.0105594.s001.tif]
