# Supplementary material for: An Enzymatic Platform for the Synthesis of Isoprenoid Precursors
Source: PLoS One. 2014 Aug 25;9(8):e105594. doi: 10.1371/journal.pone.0105594 (PMC4143292; doi:10.1371/journal.pone.0105594)
Supplement: Text S1 — (DOCX) [file pone.0105594.s003.docx]

**Supporting Information**

An Enzymatic Platform for the Synthesis of Isoprenoid Precursors

Sofía B. Rodríguez and Thomas S. Leyh

**Materials and Methods**

**Synthesis of [4-^2^H_2_]DPM**

The synthesis of [4-^2^H_2_]DPM was accomplished in two steps. First, the tautomerization of acac-CoA was used to exchange protons at the C_2_-position of the 3-oxobutyryl-moiety of acac-CoA with solvent deuterons (Fig. 3). This was accomplished by synthesizing acac-CoA in D_2_O (99%) under the following conditions: Ac-CoA (3.0 mM), acac*-*CoA thiolase (2.0 μM), MgCl_2_ (6.0 mM) and Hepes/K^+^ (50 mM) pH 8.0, T = 25 ± 2 ºC. After three hours, the reaction reached completion, ~ 70% of the Ac-CoA had been converted to acac-CoA and the methylene protons had exchanged fully with solvent (confirmed by ^1^H NMR). In the second step, acac*-*CoA thiolase was removed by ultrafiltration (10-kDa cutoff membrane) to prevent formation of unlabelled acac-CoA in the subsequent reactions. The synthesis of labeled DPM was initiated by adding the following reactants to the strained solution containing [^2^H]-acac-CoA (1.0 mM) in D_2_O: HMG-CoA synthase (4.0 μM), HMG-CoA reductase (2.0 μM), MVK (2.0 µM), PMK (1.0 µM), PK (10 U /mL), PEP (5.0 mM), NADPH (3.0 mM), Ac-CoA (1.5 mM), ATP (5.0 mM), KCl (50 mM), and β-ME (10 mM). Following 6 hr of gentle stirring at T = 25 ± 2 ºC, the reaction reached completion and > 95% the [^2^H_2_]-acac-CoA had been converted to [4-^2^H_2_]DPM. The quantitation of DPM is described above (see, *The synthesis of (R)-diphosphomevalonate*).

**The synthesis of [2, 4, 6-^2^H] DPM**

The following one-pot reaction mixture was used for this synthesis: acac*-*CoA thiolase (2.0 μM), HMG-CoA Synthase (4.0 μM), HMG-CoA reductase (2.0 μM), MVK (2.0 µM), PMK (1.0 µM), PK (10 U/mL), PEP (10 mM), [^2^H]Ac-CoA (5.0 mM), NADPH (3.5 mM), ATP (6.0 mM), MgCl_2_ (7.0 mM)*,* KCl (50 mM), β-ME (10 mM), Hepes/K^+^ (50 mM), pH 8.0, T = 25 ± 2 ºC. The reaction mixture was incubated for ~8 hrs, until reaction was completed.

**The synthesis of [6-^2^H_3_]DPM**

Mevalonolactone was linearized for enzymatic conversion to DPM by suspending 385 μmoles (50 mg) of (*R, S*)-[^2^H_3_]methyl-mevalonolactone in 200 μl of water containing five lactone-equivalents of KOH, and the solution was incubated at 37 °C for 1 h. The pH was adjusted to 7.5 with 1.0 M HCl, and Hepes/K^+^ (1.0 M, pH 7.5) as added to 50 mM. The concentration of the R-isomer (50% of the racemate) was determined by enzymatic assay (see, *Enzymatic Assays*). [6-^2^H_3_]DPM was synthesized in a one-pot-reaction under the following conditions: (*R, S*)-[6-^2^H_3_]mevalonate, MVK (2.0 µM), PMK (1.0 µM), PK (10 U/ml), ATP (5.5 mM), PEP (10 mM), β-ME (10 mM), MgCl_2_ (6.5 mM) and Hepes/K^+^ (50 mM, pH 8.0), T = 25 ± 2 ºC. The reaction achieved > 97% conversion of (*R*)-[6-^2^H_3_]mevalonate to DPM, which was quantitated as described above (see, *Enzymatic Assays*).

**Results and Discussion**

*The synthesis of [2, 4, 6,-^2^H_7_]- and [6-^2^H_3_]DPM*. The reaction conditions are detailed in *Supplementary Material*. The labeling of DPM was confirmed using ^1^H NMR (Fig. S1).

*Synthesis of [4-^2^H_2_]DPM*. ^1^H NMR confirmed that exchange was complete and occurred exclusively at the C_4_‑position of DPM (Fig. S1). It is notable that this exchange suggests the possibility of using equilibrium isotope exchange to produce Ac-CoA in which the methyl-protons have been exchanged with solvent.

A typical ^31^P NMR spectrum of synthesized DPM is shown in Figure S2. The chemical shifts and splitting patterns are consistent with literature values for the pyrophoryl-moiety [[1](#_ENREF_1)]. The purified DPM isotopomers were checked for contamination with mevalonate and/or phosphomevalonate using mevalonate and phosphomevalonate kinase (see, *Enzymatic Assays, Materials and Methods*). These contaminants are detected by coupling their phosphorylation to the oxidation of NADH [[2](#_ENREF_2), [3](#_ENREF_3)]. Contamination with either species was < 2%.

**References**

1. Gorenstein DG (1984) Phosphorus-31 NMR : principles and applications. Orlando, Fla.: Academic Press. xiv, 604 p. p.

2. Pilloff D, Dabovic K, Romanowski MJ, Bonanno JB, Doherty M, et al. (2003) The kinetic mechanism of phosphomevalonate kinase. J Biol Chem 278: 4510-4515.

3. Andreassi JL, 2nd, Dabovic K, Leyh TS (2004) Streptococcus pneumoniae isoprenoid biosynthesis is downregulated by diphosphomevalonate: an antimicrobial target. Biochemistry 43: 16461-16466.
